# EXPERIENCE OF LOW-INCOME COGNITIVELY IMPAIRED COMMUNITY-DWELLING OLDER ADULTS WITH MINDFUL MOVEMENT

**DOI:** 10.1093/geroni/igae098.2712

**Published:** 2024-12-31

**Authors:** Eunice Ojo, Ladda Thiamwong

**Affiliations:** University of Central Florida Orlando Florida, Orlando, Florida, United States; University of Central Florida, Orlando, Florida, United States

## Abstract

Falls are a common but avoidable problem among older adults. Older adults have a higher risk for falls and face many challenges to engaging in exercise-based fall prevention interventions. Practicing mindful movement contributes to gait improvement and fall prevention. However, there is limited information about the experience of older adults with mindful movement. This study aimed to explore the knowledge and experience of low-income cognitively impaired community-dwelling older adults with mindful movement. Participants aged 60 years and older with cognitive impairment were selected from a technology-based fall risk assessment study using BTracks Balance System, Fitbit, and Bioelectrical Impedance Analysis in low-income settings in Central Florida. Fourteen participants were individually interviewed, and a semi-structured interview guide was used to obtain participants’ experience with mindful movement. Individual interviews were audio-recorded, transcribed, coded, and analyzed using thematic analysis. Many codes emerged, and three major themes and sub-themes were developed including 1) knowledge of mindful movement 2) perceived benefits of mindful movement, and 3) linking mindful movement into physical activity. Most participants did not understand mindful movement but acknowledged regularly practicing careful movement to prevent falls. Participants identified fall prevention and stable mental health as benefits of mindful movement. They also described practicing mindful movement during performing physical activity including walking and dancing. There is a need to educate cognitively impaired older adults about mindful movement and encourage its practice as a vital part of daily physical activity. Future studies should focus on how to integrate mindful movement into fall prevention interventions.

